# Massive pulmonary thromboembolism combined with transient thyrotoxicosis in an 18 year old girl

**DOI:** 10.1186/s40885-020-00150-2

**Published:** 2020-09-01

**Authors:** Tong-Yoon Kim, Sang-Hyun Ihm, Ji Woong Roh, Sungmin Lim, Chan-Seok Park, Hee-Yeol Kim

**Affiliations:** Department of Internal Medicine, Bucheon St. Mary’s Hospital, College of Medicine, The Catholic University of Korea, 327, Sosa-Ro, Womni-Gu, Bucheon-Si, Gyunggi-Do 14647 Republic of Korea

**Keywords:** Pulmonary thromboembolism, Deep vein thrombosis, Thyrotoxicosis

## Abstract

**Background:**

Pulmonary thromboembolism (PTE) is thought to usually stem from deep vein thrombosis (DVT). However, evidence of DVT could not be found in many cases. Furthermore, transient thyrotoxicosis is a rare but potentially life–threatening emergency involving a systemic hypercoagulable state. We report on an 18 year-old-girl with transient thyrotoxicosis with massive PTE without DVT.

**Case presentation:**

An 18-year-old girl was admitted to the hospital with syncope. Patient had no history of trauma, any known underlying disease or oral contraceptives use. Chest computed tomography (CT) showed massive PTE in both central pulmonary arteries and diffuse goiter. However, a low extremity Doppler sonogram did not detect DVT. To manage the PTE, we administered low molecular weight heparin. On the other hands, thyroid function test indicated a state of thyrotoxicosis. In addition, patient had a partial protein S deficiency but no other immunologic abnormality. Therefore, the patient was diagnosed with massive PTE, thyrotoxicosis, and partial protein S deficiency. Patient was discharged with oral warfarin and methimazole. A follow-up echocardiogram obtained 3 months after anticoagulation therapy demonstrated normal dimensions and systolic function. After thyrotoxicosis was treated with methimazole for a month, a euthyroid state was achieved and the goiter decreased to a normal size. The methimazole was gradually tapered off and stopped at 4 months. At a 6-month follow up visit, PTE and pulmonary hypertension had disappeared but the patient still had a partial protein S deficiency. We decided to stop all medication with careful monitoring. During a 4-year follow-up period after the episode, she was asymptomatic without any evidence of recurrent systemic thromboembolism or hyperthyroidism.

**Conclusions:**

Early recognition and appropriate treatment of PTE combined with transient thyrotoxicosis were vital to preventing other complications.

## Background

Pulmonary thromboembolism (PTE) is thought to usually arise from deep vein thrombosis (DVT) [1]. However in the clinical setting, more than half of all patients do not exhibit clear signs of DVT in the presence of PTE. PTE without DVT has been explained in many ways, such as a low sensitivity of the devices used to detect DVT, rapid resolution of the condition, and development from the pulmonary trunk or cardiac tissue rather than DVT [2].

Thyrotoxic crisis is known to predispose an individual towards heart failure, atrial fibrillation, and a hypercoagulable state and represents a rare endocrine emergency with a mortality rate of 10–20%. Therefore, it is very important to recognize it early and to initiate appropriate treatment [3]. The most common underlying cause of thyrotoxicosis is Graves’ disease but transient thyrotoxicosis with a hypercoagulable state is very rare [4].

We describe a case of massive PTE without any evidence of DVT which was found to be a transient hyperthyroidism.

## Case presentation

**Fig. 1:**
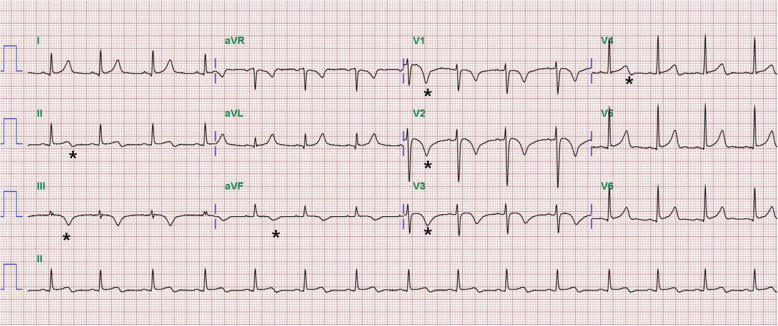
Electrocardiogram demonstrated diffuse T wave inversions (Asterisk) in the right precordial leads (V1–4) and the inferior leads (II, III, aVF)

**Fig. 2:**
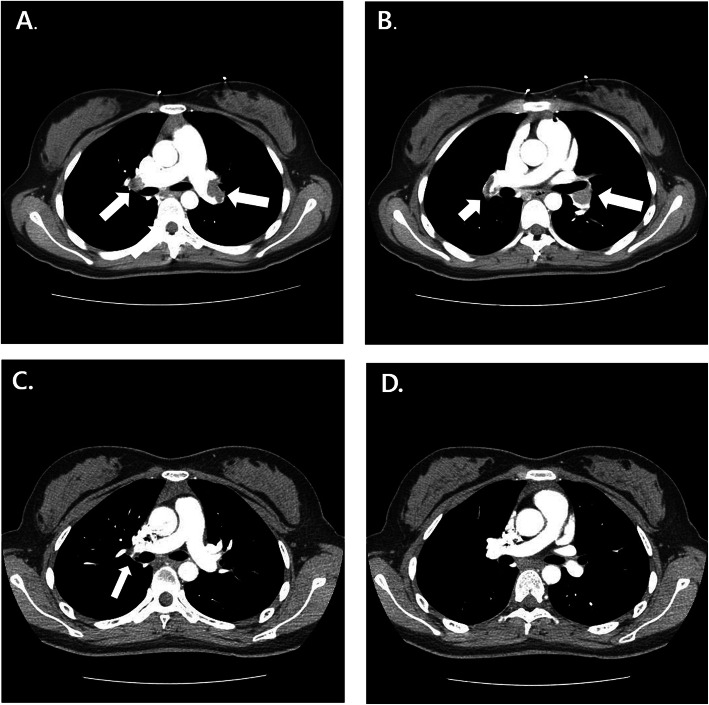
Chest computed tomography (CT) scan with contrast showing multifocal thromboemboli (White Arrow) in both pulmonary arteries (**a** and **b**). Repeated CT scan performed six months after anticoagulation therapy showing complete resolution of the thromboemboli of the main branches of both pulmonary arteries (**c** and **d**)

**Fig. 3:**
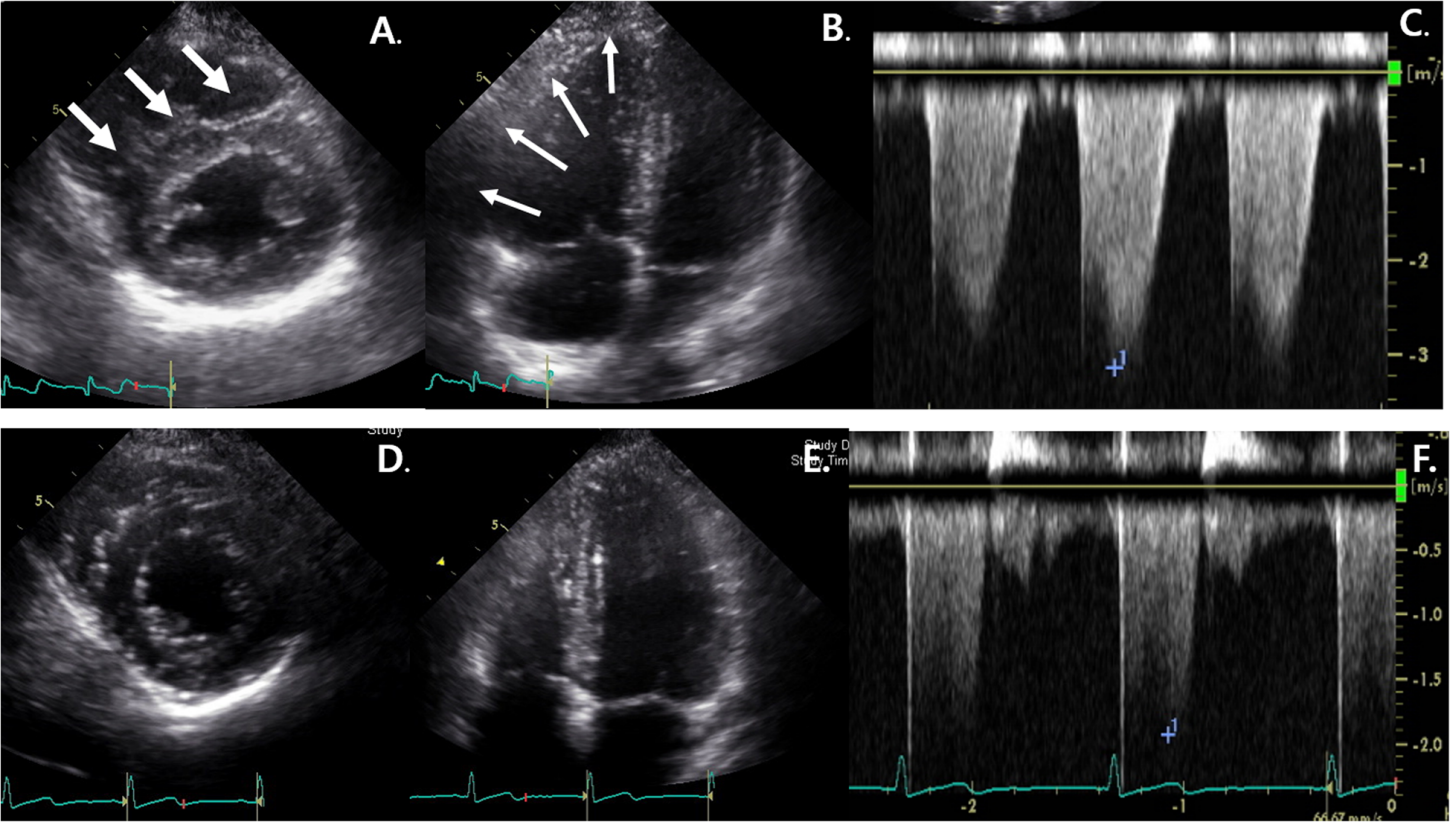
Transthoracic echocardiographic (TTE) findings. **a** Parasternal short axis view on TTE performed on admission showing flattening of the interventricular septum (D-shaped left ventricle (LV): White Arrow). **b** Apical-four-chamber view on TTE performed on admission showed marked right ventricular (RV) dilatation (White Arrow). **c** Continuous wave Doppler echocardiographic study performed on admission. Pulmonary artery systolic pressure (64 mmHg) was calculated using maximum velocity of the tricuspid regurgitation jet (Vmax = 3.3 m/sec) and estimated right atrial pressure (20 mmHg). **d** Parasternal short axis view on TTE performed 3 months after anticoagulation therapy showing normalization of RV size and disappearance of the D-shaped LV. **e** Repeated TTE performed 3 months after anticoagulation therapy showed normalization of RV cavity with improved systolic function. **f** Continuous wave Doppler echocardiographic study performed 3 months after anticoagulation therapy. Pulmonary artery systolic pressure was normalized using maximum velocity of the tricuspid regurgitation jet (Vmax = 1.9 m/sec)

**Fig. 4:**
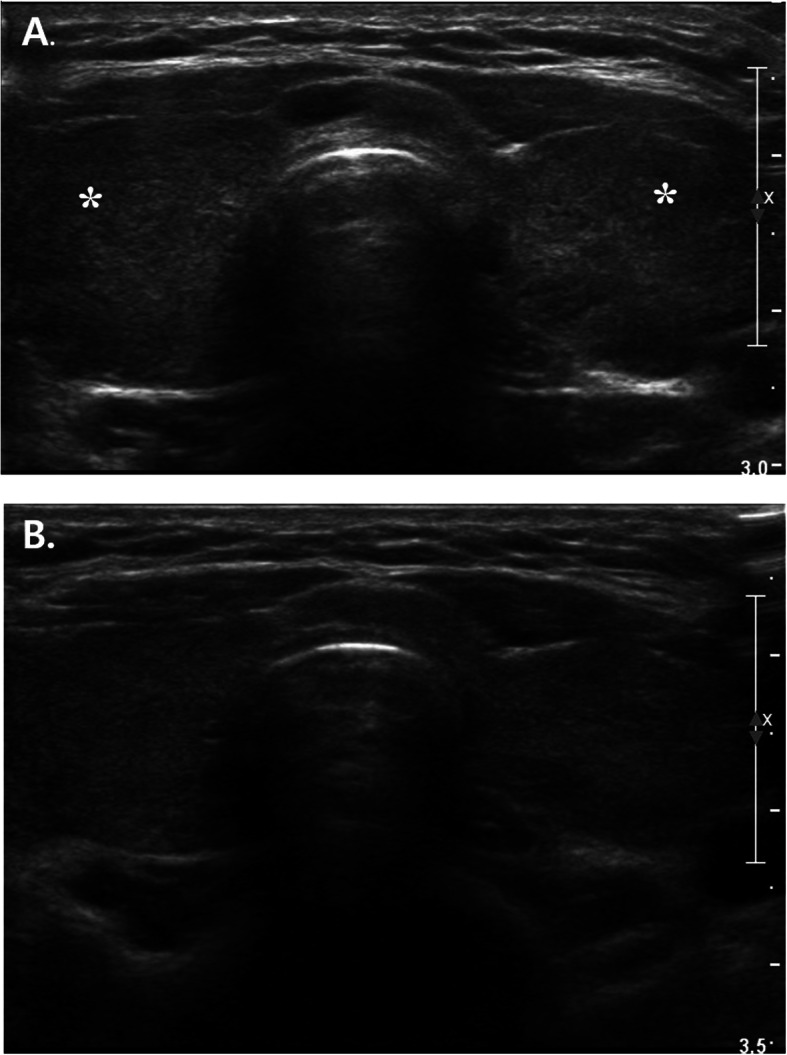
Ultrasonography of thyroid on admission (**a**) showed more hypoechoic and enlarged thyroid gland (White Asterisk) but 6-month follow-up ultrasonography (**b**) showed normal sized thyroid gland

An 18-year-old girl presented to the emergency department in our hospital with syncopal attack for 30 s. The patient had experienced palpitation, external dyspnea and chest discomfort for a week before admission. She did not have recent history of trauma, immobilization and infectious disease. She did not have any known underlying disease and was not pregnant. She has never smoked, drunken alcohol, or taken oral contraceptive. She was alert but complained mild dizziness. Her neurologic examination showed normal findings. Her vital signs were as follows: blood pressure, 70/50 mmHg; pulse rate, 110 beats/minute; temperature, 36.5oC; respiratory rate, 34/min; and oxygen saturation, 94.3% in ambient conditions. Blood studies revealed the following: white blood cell count, 11,440/μL; hemoglobin, 14.4 g/dL; non-fasting glucose, 104 mg/dL; blood urea nitrogen, 13.1 mg/dL; creatinine, 0.7 mg/dL; aspartate transaminase, 118 IU/L; and alanine transaminase, 88 IU/L. A thyroid function test indicated a state of thyrotoxicosis [T3 236.5 ng/dL (normal range, 80–200), free T4 28 pg/mL (8.9–17.9), thyroid-stimulating hormone, 0.08 mIU/L (0.17–4.05), thyrotropin binding inhibiting immunoglobulins (TBII), less than 0.3 (0 – 1 IU/L)]. Several coagulation factors were abnormal [D-dimer, 6.77 mg/L (0–0.55); protein S activity, 18% (55–123); protein S antigen, 19.2% (50–150); protein C Ag, 76% (72–160); and protein C activity, 81% (70–130)]. Other immunological workup did not reveal any evidence of connective tissue disease or antiphospholipid syndrome. An electrocardiogram showed T wave inversions in the inferior and precordial leads (Fig. 1). Chest computed tomography (CT) showed massive PTE in both central pulmonary arteries and diffuse goiter (Fig. 2a, b). However, there were no DVT in lower extremities, upper extremities and jugular veins. An echocardiogram showed severe pulmonary hypertension (right ventricular systolic pressure (RVSP) = 64 mmHg), and a dilated RV, right atrium and main pulmonary artery (Fig. 3a-c). Therefore, the patient was diagnosed with massive PTE, thyrotoxicosis, and partial protein S deficiency. To manage the PTE, we administered low molecular weight heparin for 5 days and added warfarin while adjusting the prothrombin time such that the internal normalized ratio was between 2.0 and 3.0. On the 7th day after admission, she was discharged with oral warfarin and methimazole. A follow-up echocardiogram obtained 3 months after anticoagulation therapy demonstrated normal dimensions and systolic function of the RV and disappearance of the D-shaped left ventricle (LV) (Fig. 3d-f). After thyrotoxicosis was treated with methimazole for a month, a euthyroid state was achieved and the goiter decreased to a normal size (Fig. 4). The methimazole was gradually tapered off and stopped at 4 months. At a 6-month follow up visit, PTE and pulmonary hypertension had disappeared (Fig. 2c, d) but the patient still had a partial protein S deficiency. We decided to stop all medication with careful monitoring. During a 4-year follow-up period after the episode, she was asymptomatic without any evidence of recurrent systemic thromboembolism or hyperthyroidism and had still a partial protein S deficiency.

## Discussion

Thyrotoxic crisis is a rare endocrine emergency. It typically occurs in patients with untreated or partially treated thyrotoxicosis who experience a precipitating event such as surgery, infection, or trauma. Upon admission, our patient showed features of Graves’ disease including diffuse goiter, but her TBII level was normal. TBII-seronegative patients could be discovered only 5.4% cases in Graves’ hyperthyroidism and it seemed to be less severe thyrotoxicosis and no Graves’ orbitopathy.^4^ However, after she was treated for thyrotoxicosis with methimazole for a month, a euthyroid state was achieved and the goiter was decreased to a normal size and this state has been maintained since then without any antithyroid drugs. We suggested she suffered transient thyrotoxicosis due to silent thyroiditis which is less common cause of thyrotoxicosis. In addition, thyrotoxicosis makes a hypercoagulable and hypofibirinolytic state [5]. Several pathophysiological mechanisms have been proposed to underlie the relation between thyroid hormone excess and hemostasis [5–7]. One of possible mechanisms is the direct effect on gene transcription of coagulation and fibrinolytic proteins, more specifically via the thyroid hormone receptor β [5, 6]. According to pervious large case-control study for venous thromboembolism, showed that levels of FT4 in citrated plasma within the reference range were positively associated with plasma levels of VWF and FVIII [8]. Therefore, increased free thyroxine levels cause hypercoagulability (mainly through increased levels of VWF and FVIII) and impaired fibrinolysis. Another is the activation of the autoimmune system in thyroid disease. Many thyroid diseases are immune-mediated, and other autoimmune disorders are associated with an increased risk of developing venous thromboembolism [5–7].

Protein S deficiency can result in thrombophilia, which increases the tendency to form blood clots. A deficiency in protein S activity disrupts activating protein C cleavage of Factor Va and decreases inactivation of factor Va [9]. Thrombophilia increases the risk of a systemic venous thromboembolism including PTE and DVT. In severe cases of protein S deficiency, infants develop a life-threatening blood clotting disorder, but mild protein S deficiency increases the risk of a systemic venous thromboembolism including DVT. However, it is relatively rare, only massive pulmonary embolism has been seen without evidence of DVT. Furthermore, the women with protein S deficiency seem to be at a greater risk of developing venous thromboembolism early in life (< 30 years), probably because of the use of oral contraceptives and pregnancy or puerperium. However, our patient was an 18-year-old girl without the use of oral contraceptives and pregnancy. Therefore, in our case, transient thyrotoxicosis might cause massive PTE without DVT.

## Conclusions

In our case, the patient had no history of trauma or surgery. This means that there was no chance that DVT developed because of vessel injury or hypo-motility. We suggest that the transient thyrotoxicosis may have caused a hyper-coagulated state and eventually the patient developed PTE with an underlying partial protein S deficiency. In young patients complaining of dyspnea or chest discomfort, it is worthwhile to do an echocardiogram and pulmonary angio-CT to rule out PTE in the absence of DVT. In addition, although the young patients have no thrombophilia such as protein S deficiency, transient thyrotoxicosis should be suspected.

In conclusion, the present case demonstrated a rare instance of massive PTE without any evidence of DVT which was found to be a combination of transient hyperthyroidism and partial protein S deficiency in an 18 year-old-girl. The patient had no other risk factor for a hypercoagulable state. Early detection of PTE combined with transient thyrotoxicosis using echocardiography and chest CT may be crucial in such cases, and appropriate treatment for PTE and thyrotoxicosis were vital to prevent other complications. In our case, the patient had transient thyrotoxicosis which recovered following complete remission and she is now healthy without the need for anticoagulants or anti-thyroid medications.

## Data Availability

Not applicable.

## Abbreviations

PTE: Pulmonary thromboembolism
DVT: Deep vein thrombosis
CT: Computed tomography
RV: Right ventricle
LV: Left ventricle

## References

[1] Bagot CN, Arya R (2008). Virchow and his triad: a question of attribution. Br J Haematol.

[2] van Langevelde K, Šrámek A, Vincken PWJ (2013). Finding the origin of pulmonary emboli with a total-body magnetic resonance direct thrombus imaging technique. Haematologica..

[3] Nayak B, Burman K (2006). Thyrotoxicosis and thyroid storm. Endocrinol Metab Clin N Am.

[4] Lodha A, Haran M, Frankel R (2009). Thyrotoxicosis causing arterial and venous thrombosis. Am J Med Sci.

[5] Stuijver DJF, van Zaane B, Romualdi E (2012). The effect of hyperthyroidism on procoagulant, anticoagulant and fibrinolytic factors. A systematic review and meta-analysis. Thromb Haemost.

[6] Elbers LPB, Fliers E, Cannegieter SC (2018). The influence of thyroid function on the coagulation system and its clinical consequences. J Thromb Haemost.

[7] Elbers LPB, Squizzato A, Gerdes VEA (2018). Thyroid disorders and hemostasis. Semin Thromb Hemost.

[8] Debeij J, van Zaane B, Dekkers OM (2014). High levels of procoagulant factors mediate the association between free thyroxine and the risk of venous thrombosis: the MEGA study. J Thromb Haemost.

[9] Ten Kate MK, Van Der Meer J (2008). Protein S deficiency: a clinical perspective. Haemophilia.

